# 

**Published:** 1898-06-11

**Authors:** 


					ram ausriTAju. "The standard of highest purity."?Lancet. June Uth, 1895.
CADBURY'S ^ O test CADBURY'S
COGOA T ? JFtII m? COCOA
absolutely pure. 9^ t NO drugs or alkalies used.
c
wmu un^Pi ta i
NO. 611. Vol. XXIV. Eft--] SiTDBDAT,
June 11th,1898.
"Bubscription Terns,] pB)ICE TWOPENCE.

				

